# Detection of Breast Cancer-Specific Extracellular Vesicles with Fiber-Optic SPR Biosensor

**DOI:** 10.3390/ijms24043764

**Published:** 2023-02-13

**Authors:** Yagmur Yildizhan, Kaat Driessens, Hong Shen Kevin Tsao, Robin Boiy, Debby Thomas, Nick Geukens, An Hendrix, Jeroen Lammertyn, Dragana Spasic

**Affiliations:** 1 Department of Biosystems, Biosensors Group, Katholieke Universiteit Leuven, 3001 Leuven, Belgium; 2 Laboratory of Experimental Cancer Research, Cancer Research Institute Ghent, Department of Human Structure and Repair, Ghent University, 9000 Ghent, Belgium; 3 PharmAbs, The KU Leuven Antibody Center, University of Leuven, 3000 Leuven, Belgium

**Keywords:** extracellular vesicles, biosensors, fiber-optic surface plasmon resonance, breast cancer, HER2, EpCAM

## Abstract

Extracellular vesicles (EVs) have attracted great attention as potential biomarkers for cancer diagnostics. Although several technologies have been developed for EV detection, many of them are still not applicable to clinical settings as they rely on complex EV isolation processes, while lacking sensitivity, specificity or standardization. To solve this problem, we have developed a sensitive breast cancer-specific EV detection bioassay directly in blood plasma using a fiber-optic surface plasmon resonance (FO-SPR) biosensor, previously calibrated with recombinant EVs. First, we established a sandwich bioassay to detect SK-BR-3 EVs by functionalizing the FO-SPR probes with anti-HER2 antibodies. A calibration curve was built using an anti-HER2/^B^anti-CD9 combination, resulting in an LOD of 2.1 × 10^7^ particles/mL in buffer and 7 × 10^8^ particles/mL in blood plasma. Next, we investigated the potential of the bioassay to detect MCF7 EVs in blood plasma using an anti-EpCAM/^B^anti-mix combination, obtaining an LOD of 1.1 × 10 ^8^ particles/mL. Finally, the specificity of the bioassay was proven by the absence of signal when testing plasma samples from 10 healthy people unknown to be diagnosed with breast cancer. The remarkable sensitivity and specificity of the developed sandwich bioassay together with the advantages of the standardized FO-SPR biosensor highlight outstanding potential for the future of EV analysis.

## 1. Introduction

Extracellular vesicles (EVs) hold a crucial role as mediators of cell-to-cell communication by carrying the diverse molecular cargo of their parental cells, including RNA, DNA, lipids, metabolites and proteins [1]. As such, they are involved in several physiological and pathological processes within the body, from cell maintenance to tissue regeneration, as well as tumor invasion, progression, metastasis, and even activation of immunogenic responses for cancer immunotherapy [2,3,4,5]. Their effect on cancer development and potential use as noninvasive cancer biomarkers has been continuously triggering interest among researchers, offering a great prospect for cancer diagnostics, prognostics and therapeutics [2,6]. That is why the accurate and reliable characterization and detection of EVs have become crucial to meet the growing demands of clinical applications [7].

However, EV studies remain challenging because of their inherently complex biogenesis and extensive heterogeneity in size, composition, and origin [8]. As a consequence, currently there are no specific universal sets of proteins that can be used for the accurate characterization of different EV subpopulations. When EV samples originating from different sources need to be analyzed, difficulties arise in terms of accurate comparison of data. Therefore, the International Society for Extracellular Vesicles recommends careful characterization of EV proteins to avoid: (1) overestimation of total protein concentration and (2) false assumptions about the uniform presence of proteins on the EVs (that might be caused by contamination with high-abundance matrix proteins like albumin [9,10] or as a result of EV lysis required for some analytical approaches).

Even though there are many well-established conventional methods and emerging technologies for EV characterization and detection, the absence of analytical instruments well calibrated with reference material is still a significant problem in the field [9,11]. Among the most favored conventional methods, Western blotting (WB) is the most preferred EV analysis technique that can identify the size of the different proteins and allows semiquantitative assessment of proteins of choice. The second most preferred technique in this context is enzyme-linked immunosorbent assay (ELISA) as it offers significant flexibility with respect to the bioassay formats [9]. However, while WB or ELISA may give an accurate insight for a highly purified EV population, this becomes more challenging when working directly in a complex biological fluid because of the presence of various molecules with sizes and physical properties overlapping with the EVs [12]. Consequently, the exploitation of these methods relies profoundly on the purity of the EV sample to obtain a reliable and reproducible analysis that can be transferred to clinical settings. Furthermore, both approaches are limited in their use in clinics due to lengthy preparation steps and analysis time, as well as requirement for a well-equipped facility. Besides these two, mass spectroscopy (MS) is a crucial analysis method that can achieve high-throughput, quantitative, and comparative proteomic but also lipidomic analyses of EVs [13,14]. Moreover, it can uncover the functional activities of EV cargo and their role in intercellular communication [13]. Despite these benefits, MS has several disadvantages, such as the requirement for highly purified EV samples to avoid contamination by other soluble biomolecules, which can cause aspecific signals. In addition, there is a prerequisite for peptide profiling, which entails complex processing of EVs by separating peptides via enzymatic digestion. As such, MS needs proper protein profiling, quantification, and validation through other techniques that should be calibrated with a reference material [15]. Therefore, these techniques, although conventional, still suffer from the lack of standardization prior to analyzing complex biological samples in order to ensure reproducible quality independently of the internal complexity of the measured samples.

In addition to these conventional technologies, many emerging techniques have been developed throughout the years based on different detection principles from magnetic to electrochemical and plasmonic sensing considering the great potential of EVs to be utilized for liquid biopsy and therapy of cancer. Shao et al. developed a technology for EV analysis through magnetic detection in which an on-chip micro-nuclear magnetic resonance (μNMR) detection system is integrated with immunocapture for quantitative EV detection and protein profiling [16,17]. Their study demonstrated rapid and highly sensitive detection of glioblastoma-derived circulating microvesicles, a subtype of EVs, in clinical samples. Although the detection sensitivity of μNMR surpassed the sensitivity of WB, ELISA, and nanoparticle tracking analysis (NTA) by 10^4^-, 10^3^-, and 10^2^-fold, respectively, this technology was developed using only EVs originating from cell culture or human body fluids. The development of biosensing technologies based only on specific biological samples could create bias, since it will be established considering the intrinsic features of only a certain sample, which could harm the reproducibility and reliability of the biosensor when another biological sample of different origin is analyzed. Another example of sensitive EV detection (<10^5^ EVs) are electrochemistry-based biosensors, which have even been integrated into a single iMEX (integrated magnetic–electrochemical exosome detection) platform together with magnetic enrichment, yielding fast and simplified EV analysis starting from only 10 µL of the sample [18,19]. Nevertheless, like μNMR, iMEX was also developed using only specific biological materials, such as ovarian cancer cell line-derived EVs and plasma from patients with ovarian and colorectal cancer. Consequently, the main limitation of these approaches is the lack of a standardized material during their development, which can seriously affect the reproducibility of the results.

Among various EV detection principles, surface plasmon resonance (SPR) stands out with its capacity to enable real-time and label-free kinetics and affinity measurements. Currently, there are many conventional SPR platforms, developed based on Kretschmann prism configuration or alternative approaches, such as SPR coupled with imaging (SPRi) or microscopy (SPRM), to achieve even higher throughput in EV analysis [20]. For example, SPRi enables parallel measurements dependent on the number of sensing spots and can be combined with microfluidics to support the use of fewer reagents. An SPRi biosensor was used to detect EVs isolated from various non–small cell lung cancer and normal lung cell lines with an LOD of 10^7^ EV/mL [21]. However, SPRi platforms may suffer from insufficient image resolution. In this context, SPRM technologies have been introduced for higher-resolution imaging that can even enable single-level EV detection. Yang et al. presented an SPRM platform that can detect human lung cancer cell line-derived EVs providing information regarding the size, concentration and biochemical properties of EV membrane proteins [22]. Even though these techniques enable visualization of the EVs, they are mostly suited for research purposes rather than clinical use, since the small field of view prevents parallel measurements.

**Scheme 1:**
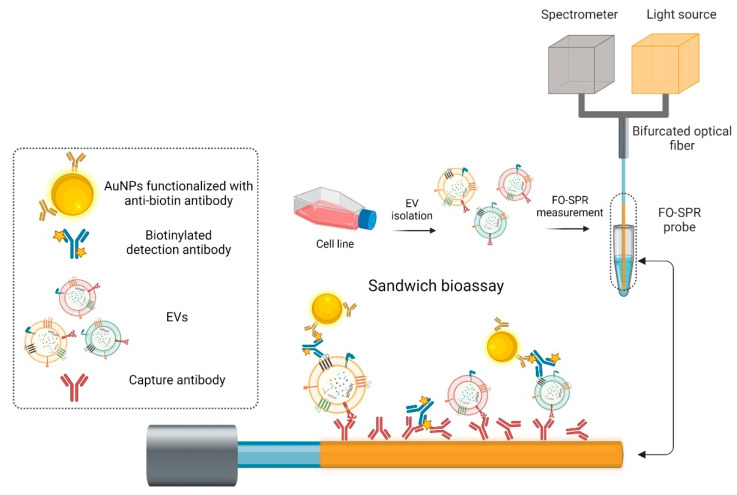
Schematic of the different steps from the FO-SPR EV detection sandwich bioassay. The EVs (originating from SK-BR-3 or MCF7 cell lines) are specifically captured by the capture antibodies immobilized on the FO-SPR surface (i.e., anti-HER2 or anti-EpCAM, respectively). Biotinylated detection antibodies (^B^anti-CD9, ^B^anti-CD63 or ^B^anti-CD81, introduced separately or as a mixture) are used to detect CD9, CD63 and CD81 tetraspanins on the EV surface. Finally, the AuNPs functionalized with anti-biotin antibodies, which recognize multiple biotin labels on the detection antibodies, are used for signal amplification. Antibodies on the FO-SPR surface as well as on the AuNPs have random orientation due to their immobilization through covalent bonds or physical adsorption, respectively. The EVs are depicted in different sizes and colors to simulate the EV heterogeneity in a biological sample. This image was created with Biorender.com (not drawn to scale).

To overcome the common limitations in the field (i.e., lack of standardization, requirement for highly purified samples, and lengthy workflows not being suitable for clinical use) that hinder the progression of EV-based diagnostics, in this study we used our fiber-optic surface plasmon resonance (FO-SPR) biosensor (commercialized by FOx Biosystems). This biosensor was previously carefully calibrated with well-characterized recombinant EVs (rEVs), i.e., biological reference materials aiming to support EV isolation and analysis, assay development, and device calibration [23,24]. Moreover, in that study we preliminary showed its potential to detect EVs in complex matrices by measuring EVs spiked at single concentration. This potential is further elaborated in this work, where we develop sensitive and specific FO-SPR bioassays for the detection of two types of breast cancer-specific EVs, both in buffer and complex biological matrix, namely, human blood plasma (Scheme 1). To achieve this, we first select two different (commonly used) breast cancer cell lines, being SK-BR-3 and MCF7, with the aim to have model systems for two breast cancer biomarkers, i.e., HER2 and EpCAM, respectively. To specifically detect EVs from both cell lines, we first screen combinations of capture and detection antibodies, which also offers us a possibility to study colocalization of proteins on EVs. Next, we use the most optimal combinations to build for the first time calibration curves for detecting EVs in 100-fold diluted blood plasma (in this case EVs originating from SK-BR-3 and MCF7 cell lines). Finally, the specificity of the established bioassays is tested with plasma samples from 10 healthy individuals as well as pooled plasma from more than 25 healthy people. Compared to other EV detection platforms, our FO-SPR sensor offers several advantages by (1) having low-cost sensor probes, (2) requiring low sample volume, (3) enabling both label-free and sandwich bioassays, and (4) being compatible with diverse complex matrices, including serum, plasma, whole blood and cell culture media [25,26].

## 2. Results and Discussion

### 2.1. FO-SPR Surface Functionalization with HER2-Specific Antibodies

**Figure 1:**
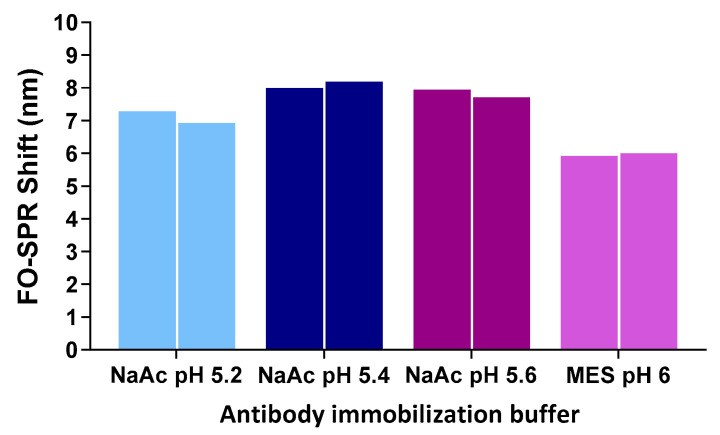
Bar graphs representing the FO-SPR shifts for the immobilization of anti-HER2 antibody using 4 immobilization buffers (10 mM NaAc buffer pH 5.2, 5.4, 5.6, and 50 mM MES buffer pH 6.0) obtained from two independent measurements.

To specifically detect EVs originating from the SK-BR-3 breast cancer cell line, FO-SPR surface was functionalized with a HER2-specific antibody, given that HER2 (human epidermal growth factor receptor 2) has a critical role in the mediation of growth and progression of breast cancer cells and is overexpressed in the SK-BR-3 cell line [27,28]. The efficiency of antibody immobilization depends on several factors, including antibody concentration, immobilization time, buffer ionic strength, and pH value. Here, 20 μg/mL was selected as the optimal antibody concentration based on saturated FO-SPR surface previously obtained for most of the tested antibodies when using COOH surface chemistry [23,29,30]. Buffers with different pH values were examined, since the isoelectric point (pI) of the selected antibody was unknown. In order to maximize the immobilization efficiency, i.e., to obtain the maximum FO-SPR shift, we screened buffers with lower ionic strength, being 10 mM sodium acetate (NaAc) buffer (pH 5.2, 5.4 and 5.6) and 50 mM MES buffer (pH 6.0). Based on this screening experiment, 10 mM NaAc buffer with pH 5.4 resulted in the highest FO-SPR shift compared to the other three buffers (Figure 1) and was selected for immobilizing anti-HER2 antibody in further experiments.

### 2.2. Development of an FO-SPR Sandwich Bioassay for Detecting SK-BR-3 EVs in Buffer

**Figure 2:**
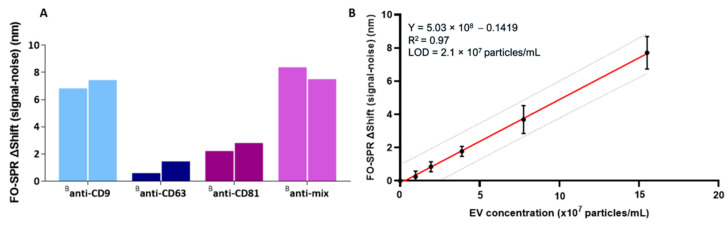
(**A**) FO-SPR sandwich bioassay established with different antibody combinations for detecting SK-BR-3 EVs in buffer. Bar graphs represent the FO-SPR shifts (obtained after subtracting the negative control, i.e., SPR signal for 0 particles/mL) by combining anti-HER2 as capture antibody with different detection antibodies (^B^anti-CD9, ^B^anti-CD63, ^B^anti-CD81 or ^B^anti-mix) for detecting SK-BR-3 EVs at 1.55 × 10^8^ particles/mL concentration (measurements shown from two independent experiments). (**B**) FO-SPR-based detection of a series of SK-BR-3 EV concentrations spiked in the detection buffer (50 mM MES pH 6, 0.01% BSA, 0.01% Tween 20) when using the anti-HER2/^B^anti-CD9 antibody combination (obtained after subtracting the negative control, i.e., SPR signal for 0 particles/mL). Simple linear regression fitting was performed by GraphPad Prism software (Version 8.0.1, GraphPad Software Inc., MA, USA). The dotted lines indicate the 95% prediction bands for a new observation. Error bars represent one standard deviation (*n* = 3).

An FO-SPR sandwich bioassay was first built in buffer in order to enable specific and sensitive detection of SK-BR-3 EVs. Starting from the FO-SPR probes functionalized with anti-HER2 antibody (as described in Section 3.4), the sandwich bioassay was developed using our previously established 2-step signal amplification approach [23]. The latter entails the implementation of biotinylated detection antibody in the first step, followed by AuNPs functionalized with anti-biotin antibody. In this context, we utilized biotinylated detection antibodies against one of the tetraspanins often used in EV research, i.e., biotinylated anti-CD9 (^B^anti-CD9), biotinylated anti-CD63 (^B^anti-CD63) or biotinylated anti-CD81 (^B^anti-CD81), either separately or an equal mixture of all three (i.e., ^B^anti-mix), since EVs have multiple copies of these proteins in their membranes [31]. All these combinations were probed in buffer with a fixed concentration of SK-BR-3 EVs (1.55 × 10^8^ particles/mL) and detection antibodies at 10 μg/mL concentration. Figure 2A depicts the FO-SPR signal shift after subtracting the negative control, the latter being the SPR shift detected in the sample without spiked EVs (i.e., 0 particles/mL). Based on these results, it was observed that using ^B^anti-CD9 or ^B^anti-mix detection antibodies generated higher SPR shifts (around 7 nm) compared to combinations with ^B^anti-CD63 or ^B^anti-CD81 detection antibodies. This suggested that both ^B^anti-CD9 and ^B^anti-mix conditions could be considered for specifically detecting SK-BR-3 EVs on the FO-SPR platform. However, anti-HER2/^B^anti-CD9 was selected as the preferred option because of the lower FO-SPR signal shifts (1.21 nm and 0.97 nm) of the negative control compared to 2.14 nm and 3.31 nm obtained when using the combination of anti-HER2/^B^anti-mix (Supplementary Materials Figure S1).

Tetraspanins have been described as a superfamily of membrane proteins, including adhesion, signaling and adaptor proteins, that are highly organized and regulate various cell signaling pathways that affect various biological processes [32,33]. This experiment revealed how biomarker selection could affect the final signal shift due to the heterogeneity of the tetraspanin expression profile, even originating from the same EV subpopulation [33]. Our results might further build on the previously reported involvement of CD9 tetraspanin in breast cancer invasiveness and metastases in several studies with high CD9 expression levels [34].

The combination of anti-HER2/^B^anti-CD9 was exploited for building a calibration curve by spiking in buffer different SK-BR-3 EV concentrations ranging from 9.7 × 10^6^ to 1.55 × 10^8^ particles/mL and also including a negative control without any EVs—0 particles/mL. The obtained FO-SPR signal shifts after subtracting the negative control (*n* = 3) were plotted as a function of SK-BR-3 EV concentration (Figure 2B). The calibration curves showed that the FO-SPR biosensor can detect SK-BR-3 EVs over the entire tested concentration range with a limit of detection (LOD) of 2.1 × 10^7^ particles/mL. The results indicated that the obtained LOD was 1.5-fold lower than the previously reported LOD value of rEVs (3.13 × 10^7^ particles/mL) detected in buffer using FO-SPR technology [24]. This improvement can be related to several factors, such as (1) strong affinity of selected anti-HER2 antibody against isolated SK-BR-3 EVs, (2) the number of HER2 oncogenic proteins on the EVs, and (3) the difference in number and distribution of general EV biomarkers, such as CD9, CD63, and CD81, between rEVs and SK-BR-3 EVs.

### 2.3. Development of an FO-SPR Sandwich Bioassay for Detecting SK-BR-3 EVs in Plasma

**Figure 3:**
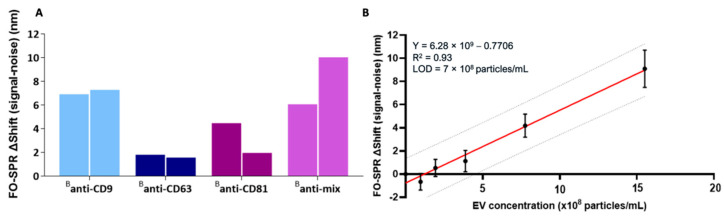
(**A**) FO-SPR sandwich bioassay with different antibody combinations for detecting SK-BR-3 EVs in 100-fold diluted pooled plasma. Bar graphs represent the FO-SPR shifts from two independent measurements (after subtracting the negative control, i.e., SPR signal for 0 particles/mL) obtained by combining anti-HER2 capture antibody with different detection antibodies (^B^anti-CD9, ^B^anti-CD63, ^B^anti-CD81 or ^B^anti-mix) for detecting SK-BR-3 EVs at 1.55 × 10^9^ particles/mL concentration. (**B**) FO-SPR-based detection of a series of SK-BR-3 EV concentrations in 100-fold diluted pooled plasma when using anti-HER2/^B^anti-CD9 antibody combination (obtained after subtracting the negative control, i.e., SPR signal for 0 particles/mL). Simple linear regression fitting was performed by GraphPad Prism software. The dotted lines indicate the 95% prediction bands for a new observation. Error bars represent one standard deviation (*n* = 3).

To further test the potential of our FO-SPR anti-HER2/^B^anti-CD9 bioassay established in buffer, SK-BR-3 EVs were spiked in 100-fold diluted pooled plasma. In this context, we first reexamined the antibody combinations by using anti-HER2 as the capture antibody together with ^B^anti-CD9, ^B^anti-CD63, ^B^anti-CD81 or ^B^anti-mix as the detection antibody. The plasma samples were spiked with an order of magnitude higher EV concentration (1.55 × 10^9^ particles/mL) compared to the experiments in buffer to ensure EVs will be detected in such a complex sample. As per Figure 3A, 7 nm signal shifts were obtained after subtracting the negative control when using ^B^anti-CD9, which matched previous results obtained in buffer (Figure 2A). Although the combination with ^B^anti-mix gave a higher signal shift in one of the repetitions (10.10 nm), the poor reproducibility of this condition (mainly due to the usage of ^B^anti-CD81 antibody) together with larger signal shifts obtained from the negative control (Supplementary Materials Figure S2) made it a less favorable condition, similar to the results obtained in buffer.

Consequently, we selected the anti-HER2/^B^anti-CD9 antibody combination to build a calibration curve in 100-fold diluted pooled plasma by spiking a series of SK-BR-3 EVs concentrations, ranging from 9.7 × 10^7^ to 1.55 × 10^9^ particles/mL (including the negative control). As described in the previous section, the calibration curves were fitted, revealing that the FO-SPR biosensor could detect SK-BR-3 EVs throughout the tested concentration range with an LOD value of 7 × 10^8^ particles/mL (Figure 3B).

The LOD of the bioassay decreased by threefold compared to detection in buffer. This might be due to the effect of the human blood plasma matrix, which contains approximately 60 to 80 mg/mL of proteins [35] and consequently, the aspecific interference of non-EV-related proteins with the fiber surface. Nevertheless, the obtained LOD was approximately 10^3^ times lower than the reported concentration of EVs in plasma of cancer patients [16], demonstrating considerable potential of the established FO-SPR bioassay for sensitive EV analysis. Moreover, while surpassing the reported LOD values from a number of technologies, like WB (10^5^-fold), ELISA (10^4^-fold), NTA (10^3^-fold) and µNMR (10-fold), our FO-SPR biosensor was 100 times less sensitive than the iMEX technology [17,18]. However, it should be noted that the iMEX technology, similar to the µNMR, detected only CD63-positive EV subpopulations without prior calibration, while the FO-SPR biosensor was previously calibrated with rEVs for reproducible and reliable detection independent from the targeted EV subpopulation [23]. Furthermore, the established FO-SPR biosensor with HER2-positive EV bioassay can potentially be used to detect EVs directly from diluted human blood plasma samples without prior EV isolation.

### 2.4. Expanding the Established FO-SPR Sandwich Bioassay towards MCF7 EVs

**Figure 4:**
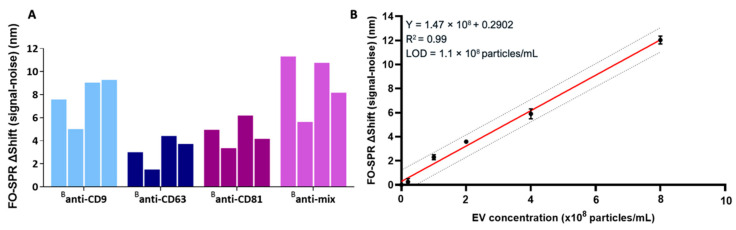
(**A**) FO-SPR sandwich bioassay with different antibody combinations for detecting MCF7 EVs in 100-fold diluted pooled plasma. Bar graphs represent the FO-SPR shifts (after subtracting the negative control, i.e., SPR signal for 0 particles/mL) obtained from four independent measurements by combining anti-EpCAM capture antibody with different detection antibodies (^B^anti-CD9, ^B^anti-CD63, ^B^anti-CD81 or ^B^anti-mix) for detecting MCF7 EVs at 1 × 10^9^ particles/mL concentration. (**B**) FO-SPR-based detection of a series of MCF7 EV concentrations when using anti-EpCAM/^B^anti-mix antibody combination in 100-fold diluted pooled plasma (obtained after subtracting the negative control, i.e., SPR signal for 0 particles/mL). Simple linear regression fitting was performed by GraphPad Prism software. The dotted lines indicate the 95% prediction bands for a new observation. Error bars represent one standard deviation (*n* = 3).

To test the applicability of the established FO-SPR sandwich bioassay, we selected another breast cancer-specific EV biomarker, namely EpCAM (epithelial cell adhesion molecule, type I transmembrane glycoprotein), to detect EVs isolated from an MCF7 breast cancer line well known to overexpress EpCAM [36]. Anti-EpCAM (20 μg/mL) was immobilized as capture antibody on the FO-SPR surface using buffers with lower ionic strength, i.e., 10 mM NaAc buffer (pH 5.2, 5.4 and 5.6) and 50 mM MES buffer (pH 6.0) with the aim of obtaining the maximum FO-SPR shift (nm). Based on the results shown in Supplementary Materials Figure S3, 10 mM NaAc buffer pH 5.6 was selected for immobilizing anti-EpCAM since it gave the highest FO-SPR shifts (8.16 nm and 8.28 nm) compared to other conditions. Subsequently, MCF7 EVs (1 × 10^9^ particles/mL) were spiked in 100-fold diluted pooled plasma. Similar to HER2-specific bioassay, here we also examined different antibody combinations with anti-EpCAM as capture antibody and ^B^anti-CD9, ^B^anti-CD63, ^B^anti-CD81 or ^B^anti-mix as detection antibodies. Based on the results presented in Figure 4A, combinations with ^B^anti-CD9 and ^B^anti-mix gave the overall highest signal shifts after subtracting the negative control (i.e., 0 particles/mL). However, as shown in Supplementary Materials Figure S4, the negative control signal of ^B^anti-mix was between 0.31 nm and 0.88 nm and distinctly lower than when used with anti-HER2 as capture antibody (Supplementary Materials Figure S2). That is why we selected the combination of anti-EpCAM/^B^anti-mix for building a calibration curve in 100-fold diluted plasma.

The combination of anti-EpCAM/^B^anti-mix was further used to build a calibration curve for MCF7 EVs spiked in 100-fold diluted pooled plasma with a series of EV concentrations, ranging from 2 × 10^7^ to 8 × 10^8^ particles/mL (including the negative control). The obtained average FO-SPR shifts (*n* = 3) were plotted as a function of EV concentrations (Figure 4B). The calibration curve showed that the FO-SPR biosensor detected MCF7 EVs with an LOD value of 1.1 × 10^8^ particles/mL, comparable to the LOD obtained from SK-BR-3 EV detection in 100-fold diluted human blood plasma.

Although the human body is abundant with EVs, the quantity of cancer-specific EVs in human blood is an undetermined variable that depends on numerous factors, such as the state of the disease, age and gender of the patients, and the applied treatment, among others. Even though the achieved LOD values are very promising compared to the conventional techniques, further improvements are required to reach higher sensitivity to utilize FO-SPR biosensor for real clinical settings. Avenues for further research might include investigating alternative antibodies with higher affinities towards EV surface proteins or other surface chemistries, such as NTA-SAM [25], to establish an organized surface through oriented antibody immobilization.

### 2.5. Testing FO-SPR Bioassays’ Specificity across Plasma Samples from Healthy Donors

**Figure 5:**
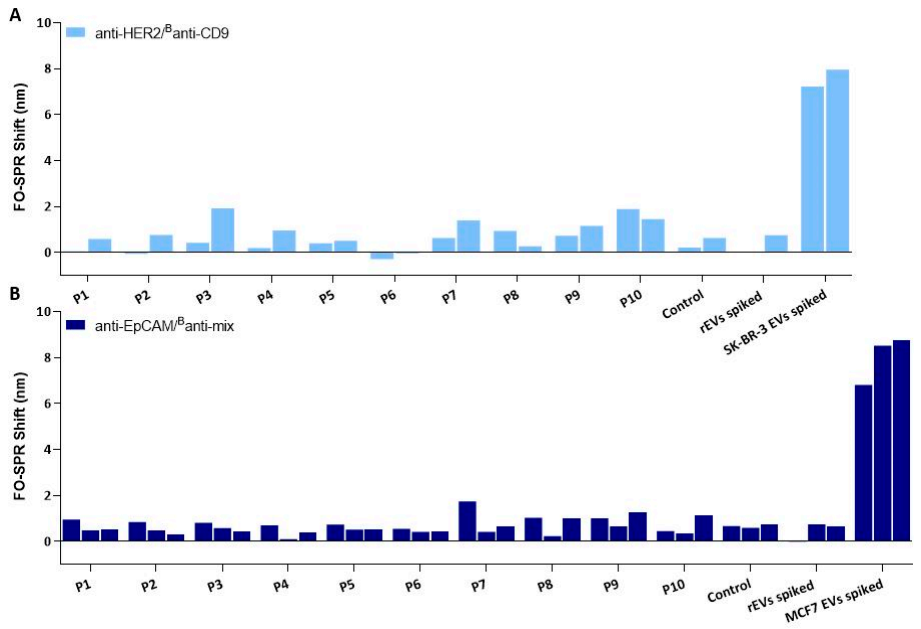
Specificity testing of the FO-SPR bioassays in 100-fold diluted plasma using (**A**) anti-HER2/^B^anti-CD9 and (**B**) anti-EpCAM/^B^anti-mix antibody combinations. P1 to 10 are plasma samples of 10 individual healthy donors unknown to be diagnosed with breast cancer. Control is 100-fold diluted pooled plasma. Two additional controls represent (1) rEVs spiked in 100-fold diluted pooled plasma and (2) SK-BR-3 (in panel (**A**) or MCF7 EVs (in panel (**B**) spiked in 100-fold diluted pooled plasma. For panel (**A**), two independent measurements were performed for all the samples, whereas the number of repetitions was three for panel (**B**) (although some of the obtained values are close to zero and thus barely visible).

In the final step, we evaluated the specificity of the established FO-SPR anti-HER2/^B^anti-CD9 and anti-EpCAM/^B^anti-mix bioassays and their potential to be used for the detection of breast cancer EVs in patient samples. This was done by running the two sandwich bioassays in the 100-fold diluted plasma from 10 individual healthy donors unknown to be diagnosed with breast cancer (P1–P10, Figure 5). As can be seen from Figure 5A,B, the SPR shift remained for all the samples well below 2 nm. Moreover, the obtained signal was similar to the one from 100-fold diluted pooled plasma (control), as well as from rEVs spiked in 100-fold diluted pooled plasma (1 × 10^9^ particles/mL), which do not express any of the breast cancer biomarkers [24]. Contrary to this, shifts of approximately 8 nm were obtained when SK-BR-3 and MCF7 EVs were spiked in 100-fold diluted pooled plasma (1 × 10^9^ particles/mL), demonstrating that this bioassay is specific to EVs that express HER2 or EpCAM proteins on their surface when anti-HER2 or anti-EpCAM were used as capture antibodies, respectively. These results support our established bioassay being not only sensitive but also highly specific to the EVs of interest. The low negative control signal detected in healthy donors highlights the potential of reliable direct detection of cancer-specific EVs in human blood plasma.

## 3. Materials and Methods

### 3.1. Reagents and Antibodies

All buffer reagents were obtained from Sigma-Aldrich (Bornem, Belgium), unless stated otherwise, and were made using deionized water purified with the Milli-Q Plus system (Millipore, Marlborough, MA, USA). The following suppliers were used for purchasing the buffer reagents: (1) AppliChem GmbH (Darmstadt, Germany) for Tween 20, (2) VWR (Leuven, Belgium) for ethanol, hydrochloric acid and sodium hydroxide, (3) Thermo Fisher Scientific (Erembodegem, Belgium) for superblock buffer, acetone and N-hydroxysuccinimide (NHS), (4) Merck Life Science (Hoeilaart, Belgium) for M N-(3-dimethylaminopropyl)-N′-ethylcarbodiimide hydrochloride (EDC), (5) GERBU Biotechnik GmbH (Heidelberg, Germany) for COOH-SAM and (6) BBI Solutions (Cardiff, UK) for AuNPs, conjugated with goat anti-biotin antibody, of 40 nm diameter and optical density (OD) of 10.

In this study, we used EV-validated antibodies previously characterized through various quality-control tests, such as WB, flow cytometry, ELISA, and FO-SPR technology, as demonstrated in our previous study [23]. Mouse monoclonal EpCAM-specific antibody (anti-EpCAM, Cat. no: 324202), as well as biotinylated anti-CD63 (Cat. no: 353018), biotinylated anti-CD9 (Cat. no: 312110) and biotinylated anti-CD81 (Cat. no: 349514) antibodies (^B^anti-CD9, ^B^anti-CD63 and ^B^anti-CD81, respectively) were obtained from Biolegend (ImTec Diagnostics, Antwerp, Belgium). HER2-specific antibody (anti-HER2, 4D5), which is a murine IgG1 equivalent of trastuzumab, was obtained from PharmAbs, the KU Leuven Antibody Center (Leuven, Belgium) [37]. All antibody concentrations are indicated in Section 3.4.

Pooled plasma was obtained from more than 25 healthy donors, and plasma of 10 random healthy donors, unknown to be diagnosed with breast cancer, was recruited at the Laboratory for Thrombosis Research (KU Leuven, Campus Kulak Kortrijk, Belgium) with a signed informed consent form. Six tubes per donor (±35 mL blood per donor) were collected using BD vacutainer trisodium citrate tubes (BD 366575, BD, Temse, Belgium), and all the tubes were pooled together into several 50 mL tubes. Later, they were centrifuged for 15 min at 2200 rpm, and plasma was collected with a sterile pipette (612-1685, VWR, Leuven, Belgium) in a sterile container on ice. After processing all blood samples, the pooled plasma was put in a warm water bath at 37 °C for 7 min to be aliquoted afterwards to 10 mL in 15 mL tubes and stored at −80 °C.

### 3.2. Isolation and Characterization of SK-BR-3, MCF7 and rEVs

SK-BR-3, MCF7 and rEVs [23,24] were separated from the culture medium of SK-BR-3, MCF7 (both originating from human breast cancer) and HEK293T (human embryonic kidney) cell lines, respectively, using OptiPrep™ density gradient (Alere Technologies AS, Oslo, Norway) as previously described [25,38]. A short description of characterization and isolation of rEVs and OptiPrep™ density gradient is given in Supplementary Materials. Aliquots of EVs were stored at −80 °C until further use and thawed carefully on ice just before the analysis.

The EV solutions were diluted in phosphate-buffered saline (PBS) buffer to a final volume of 800 μL for NTA [39]. The concentration and particle size distribution of EV samples were identified using a NanoSight LM10 (Malvern Instruments, Worcestershire, UK) configured with a 405 nm laser and connected to an sCMOS camera with the detection threshold set between 4 to 5 for recording. Six videos of 30 s were taken to calculate particle size and concentration distributions using NanoSight NTA analytical software (version 2.3, Nanosight Ltd., Wiltshire, UK) as presented in Supplementary Figure S5.

### 3.3. FO-SPR Biosensor and Manufacturing of FO-SPR Probes

A benchtop FO-SPR biosensor, introduced by our group and commercialized by FOx Biosystems (Diepenbeek, Belgium), was used to perform FO-SPR bioassays for EV detection. FO-SPR probes were prepared manually for each experiment as previously described [40,41]. In summary, a final length of 4.3 cm of FO-SPR probes was cut from a multimode optical fiber (TEQS, Thorlabs, Munich, Germany) with a core diameter of 400 μm and endings were stripped and cleaned, leaving 0.6 cm on the sensor side. Later, a thin layer (~50 nm) of gold was sputtered using a sputter coater (Quorum Q150T ES, Quorum Technologies, East Sussex, UK). Gold-coated FO-SPR probes were functionalized at 4 °C in a 0.1 mM ethanol/COOH SAM solution (volume ratio of 9:1) for 2 days. Finally, just before the experiment, the probes were washed with ethanol to remove any unbound material and used immediately.

### 3.4. FO-SPR Sandwich Bioassay for Detection of EVs in Buffer and Plasma

After the functionalization of the FO-SPR probes with COOH SAM, they were immersed in 0.4 EDC/0.1 M NHS solution dissolved in 50 mM MES buffer at pH 6 for 15 min for the activation of COOH groups on the fiber surface. Then, breast cancer EV-specific capture antibodies (i.e., anti-HER2 or anti-EpCAM) diluted in different buffers as specified throughout this study (i.e., 10 mM NaAc buffer at pH 5.2, 5.4 and 5.6, and 50 mM MES buffer at pH 6) were bound covalently to the activated COOH groups at a concentration of 20 μg/mL with shaking at 200 rpm, for 900 s. Afterwards, the FO-SPR probes were immersed consecutively in blocking buffers: superblock (300 s), 1 M ethanolamine (600 s) at pH 8, and again in superblock (300 s) to minimize the aspecific binding.

The biofunctionalized FO-SPR probes were then introduced to the detection buffer (50 mM MES pH 6 supplemented with 0.01% BSA and 0.01% Tween 20) or plasma samples diluted 100-fold in the detection buffer. Next, they were dipped into samples containing SK-BR-3 (9.7 × 10^6^ to 1.55 × 10^8^ particles/mL spiked in detection buffer or 9.7 × 10^7^ to 1.55 × 10^9^ particles/mL spiked in 100-fold diluted plasma) or MCF7 EVs (2 × 10^7^ to 8 × 10^8^ particles/mL spiked in 100-fold diluted plasma), including 0 particles/mL as a negative control for 20 min to record the real-time, label-free binding of EVs. In order to achieve signal amplification, the FO-SPR probes were subsequently immersed in the same detection buffer or 100-fold diluted plasma with 10 μg/mL concentration of biotinylated detection antibodies (^B^anti-CD9, ^B^anti-CD63, ^B^anti-CD81 or ^B^anti-mix) for 900 s at 200 rpm.

Finally, the FO-SPR probes were reintroduced into the detection buffer or 100-fold diluted plasma, followed by immersion into the PBS with 0.5% of BSA to obtain a baseline signal for the next step with AuNPs functionalized with goat anti-biotin antibody. These AuNPs were prepared, prior to their use in the FO-SPR bioassay, by centrifugation at 5000 rpm for 30 min at 4 °C. After removing the supernatant, AuNPs were resuspended in PBS with 0.5% of BSA with 1:10 dilution ratio to obtain an OD of 1. FO-SPR probes were immersed in 150 μL solution of AuNPs for 1 h. All the steps of EV detection bioassay were performed at room temperature.

### 3.5. Data Analysis

The obtained data were recorded and further processed using custom-built software developed by FOx Biosystems Ltd. (Diepenbeek, Belgium). The calibration curves were obtained by plotting the obtained FO-SPR shifts—after subtracting the negative control signal at 0 particles/mL—as a function of the different EV concentrations. A linear regression curve was fitted using GraphPad Prism software. The LOD values were calculated as EV concentrations (particles/mL) corresponding to the sum of the respective blank signal and three times the standard deviation of the blank signal.

## 4. Conclusions

This study reported on the development of a breast cancer-specific EV detection bioassay directly in blood plasma using the FO-SPR biosensor, which was previously calibrated with rEVs as a reference material [23]. For the first time, full calibration curves were established for detecting EVs with the FO-SPR bioassay, which allowed us to determine the LODs of 7 × 10^8^ particles/mL and 1.1 × 10^8^ particles/mL when using anti-HER2/^B^anti-CD9 and anti-EpCAM/^B^anti-mix as antibody combinations, respectively. Moreover, because of the implemented sandwich bioassay format, information regarding the colocalization of EV surface proteins could be obtained using FO-SPR biosensor. Cancer-specific EVs were differentiated from those originating from healthy cells as long as a cancer-specific biomarker was present. Previously, the colocalization of EV surface biomarkers was obtained either by (1) imaging strategies that require expensive equipment, while being restricted by the availability of the fluorescent labels/imaging channels or (2) conventional ELISA that requires lengthy preparation steps and processes that are highly dependent on sample purity for EV analysis. However, the FO-SPR biosensor offers a possibility to study colocalization of proteins on EVs while enabling real-time detection directly in the crude samples in a short time-to-result manner. Finally, the time to result of FO-SPR detection bioassay was further shortened for cancer-specific EV detection (compared to the previous work [23]), resulting in 2 h and 40 min, leaving additional room for improvements. These capabilities of the bioassay combined with essential features of the FO-SPR biosensor reveal the great potential of this technology to be used as a standardized diagnostic tool and significantly contributing to the EV research field.

## Data Availability

Not applicable.

## Supplementary Materials

The following supporting information can be downloaded at https://www.mdpi.com/article/10.3390/ijms24043764/s1.
