# Supplementary material for: Detection of Breast Cancer-Specific Extracellular Vesicles with Fiber-Optic SPR Biosensor
Source: Int J Mol Sci. 2023 Feb 13;24(4):3764. doi: 10.3390/ijms24043764 (PMC9966403; doi:10.3390/ijms24043764)
Supplement: Supplementary file 1 [file ijms-24-03764-s001.zip › ijms-2191628-supplementary.pdf]

International Journal of *Molecular Sciences*

MDPI

Article

Detection of Breast Cancer-Specific Extracellular Vesicles with Fiber-Optic SPR Biosensor

Yagmur Yildizhan [1], Kaat Driessens [1], Hong Shen Kevin Tsao [1], Robin Boiy [2], Debby Thomas [3], Nick Geukens [3], An Hendrix [2], Jeroen Lammertyn [1,*] and Dragana Spasic [1]

[1] Department of Biosystems, Biosensors Group, Katholieke Universiteit Leuven, 3001 Leuven, Belgium
[2] Laboratory of Experimental Cancer Research, Cancer Research Institute Ghent, Department of Human Structure and Repair, Ghent University, 9000 Ghent, Belgium
[3] PharmAbs, The KU Leuven Antibody Center, University of Leuven, 3000 Leuven, Belgium
* Correspondence: jeroen.lammertyn@kuleuven.be; Tel.: +32-16-32-14-59

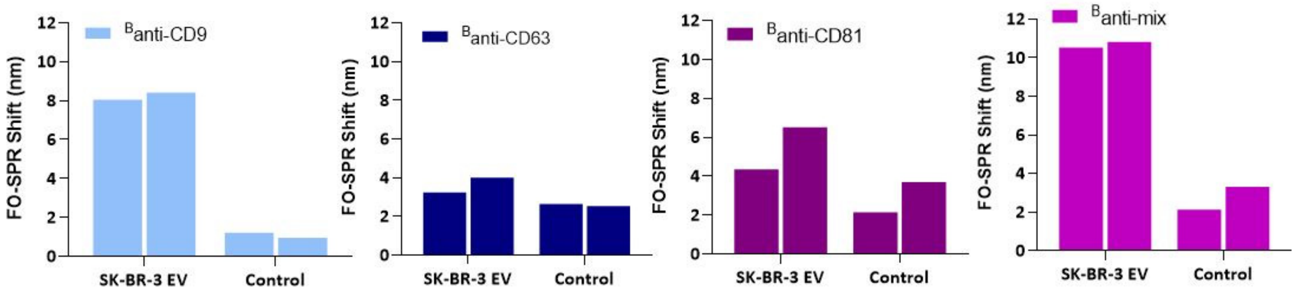

Figure S1. FO-SPR sandwich bioassay with different antibody combinations for detecting SK-BR-3 EVs in buffer. Bar graphs represent the FO-SPR shifts obtained from two independent measurements by combining anti-HER2 capture antibody with different detection antibodies (^{B}anti-CD9, ^{B}anti-CD63, ^{B}anti-CD81 or ^{B}anti-mix) for detecting SK-BR-3 EVs at 1.55 × 10^8 particles/mL concentration. Controls were performed for each antibody combination without SK-BR-3 EVs.

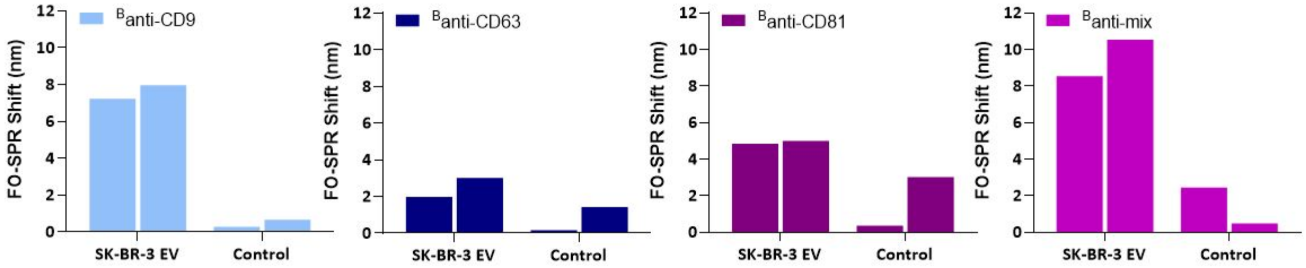

Figure S2. FO-SPR sandwich bioassay with different antibody combinations for detecting SK-BR-3 EVs in plasma. Bar graphs represent the FO-SPR shifts obtained from two independent measurements by combining anti-HER2 capture antibody with different detection antibodies (^{B}anti-CD9, ^{B}anti-CD63, ^{B}anti-CD81 or ^{B}anti-mix) for detecting SK-BR-3 EVs at 1.55 × 10^9 particles/mL concentration. Controls were performed for each antibody combination without SK-BR-3 EVs.

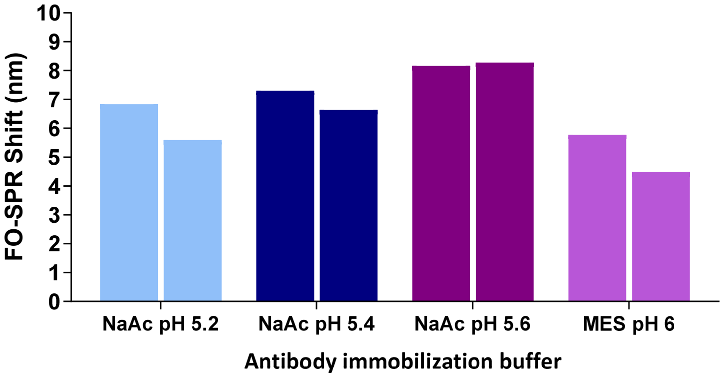

Figure S3. Bar graphs representing the FO-SPR shifts obtained from two independent measurements for the immobilization of anti-EpCAM antibody using 4 different immobilization buffers (10 mM NaAc buffer pH 5.2, 5.4, 5.6 and 50 mM MES buffer pH 6.0).

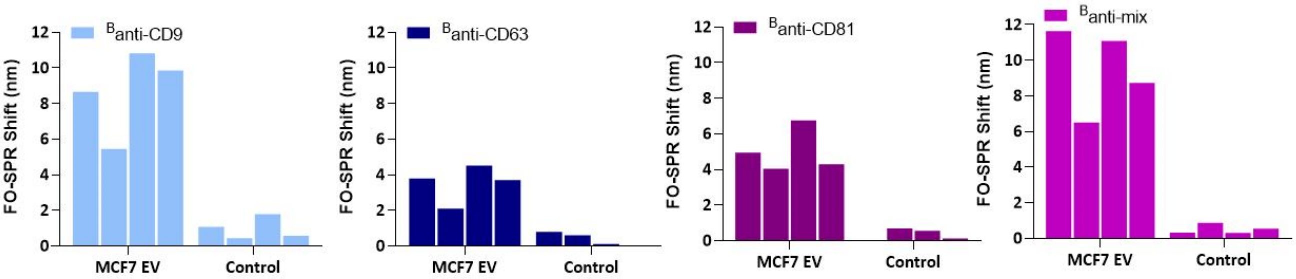

Figure S4. FO-SPR sandwich bioassay with different antibody combinations for detecting MCF7 EVs in plasma. Bar graphs represent the FO-SPR shifts obtained from four independent measurements by combining anti-EpCAM capture antibody with different detection antibodies (^{B}anti-CD9, ^{B}anti-CD63, ^{B}anti-CD81 or ^{B}anti-mix) for detecting MCF7 EVs at 2 × 10^9 particles/mL concentration. Controls were performed for each antibody combination without MCF7 EVs.

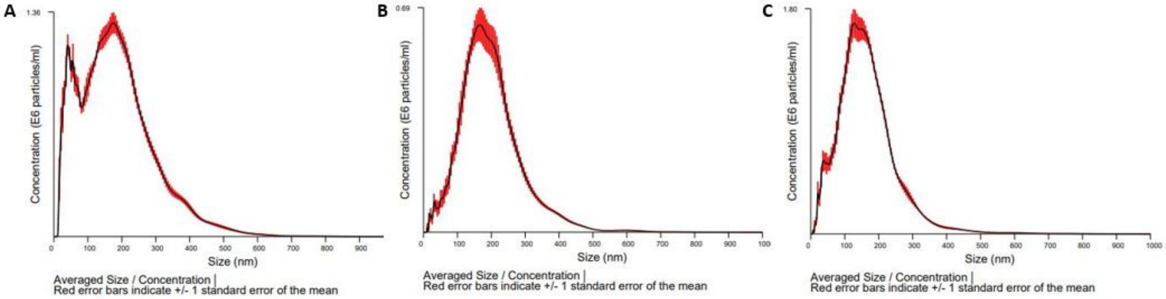

Figure S5. NTA batch report demonstrating the size and concentration distribution of **A)** SK-BR-3 EVs, **B)** MCF7 EVs and **C)** rEVs.

Recombinant extracellular vesicles (rEVs)

rEVs are produced in a well-characterized HEK293T cell culture model transiently transfected with retroviral gag polyprotein, C-terminally fused to EGFP (enhanced green fluorescent protein). While the detailed isolation and characterization process can be find in literature [1], shortly, rEVs originate from hijacking of HIV-1 gag polyprotein into the ESCRT (endosomal sorting complex required for transport) pathway, responsible for the release of EVs, and inserts itself in membrane areas with similar lipid/protein character-

istics as EV budding areas. The nanometer-sized immature virus like particles, named rEVs, surrounded by a lipid bilayer and enriched for gag molecules and EV-associated proteins (Alix, TSG101, flotillin-1, syntenin-1 and tetraspanins: CD9, CD63, CD81) are obtained after the isolation from cell culture medium. rEVs used in this paper have been previously compared to different EVs (e.g. medium conditioned by breast cancer (MCF7, 4T1) or mock transfected HEK293T cell lines) proving they have similar buoyant density, size distribution, morphology, RI, zeta potential and molecular patterns (proteins and lipids) [1]. Moreover, the gag-EGFP fusion protein enables sensitive and differential trackability of rEVs and makes them ideal biological reference materials to be used for quality control, data normalization, standardization of bioassay development and calibration of EV isolation and characterization techniques, which is vital for future of EV-based biomedical applications.

OptiPrep™ density gradient

A top-loading OptiprepTM density gradient (ODG, Alere Technologies AS, Oslo, Norway) was utilized to isolate EVs from cell culture medium (CCM) of SK-BR-3 and MCF7 cell culture. Optiprep stock solution contains 60% iodixanol (w/v), with 1.320 g/mL density, while EVs have a density of 1.08-1.22 g/mL in iodixanol. Gradient solutions were produced by diluting 5%, 10%, 20% and 40% working solutions with homogenization medium (0.25 M sucrose, 1 mM EDTA, 60 mM Tris-HCl (pH 7.4)). 1 mL of CCM was added on top of the ODG and the tubes were centrifuged at 100.000 x g at 4 °C for 18 hours with minimal deacceleration (SW 32.1 rotor, Beckman Coulter). 1 mL gradient fractions were collected starting from the top of ODG. The 9th and 10th fractions were pooled and pelleted to remove iodixanol. The pooled fractions were added to 14 mL of sterile PBS and centrifuged at 100.000 x g, 4 °C for 3 hours to pellet EVs. The supernatant was removed until ± 30 µL of the volume was retained containing the EVs. Lastly, the pellet was resuspended in 100 µL fresh PBS.

FO-SPR biosensor and manufacturing of FO-SPR probes

The FO-SPR platform allows simultaneous and completely automated measuring of 4 samples through 4 separate FO-SPR probes. These probes are connected through a bifurcated fiber to 4 different broad-spectrum white light LED sources and spectrophotometers (USB4000, Ocean Insight, FL, USA). The bifurcated fiber enables white light to travel through the sensor tip, covered with a 50 nm gold layer, where it is reflected back to the spectrophotometer. An SPR signal is generated in this gold layer where the light interacts with the surface of the optical fiber. A biomolecular binding event or any alteration in the refractive index on the outside of the gold layer interrupts surface plasmons, changing the resonance conditions and shifts the resonance wavelength. This working principle allows many biomolecular interactions to be monitored in real time.

The optical assembly of the device is protected and covered by a black container. Before starting the experiment, the sequence of dipping the FO-SPR probes into different solutions was loaded into the custom-made software, while the PCR tubes (VWR international, Belgium), filled with different buffers and reagents, were placed in the system tube holder. Next, the functionalized probes were attached to the FO-SPR sensor and parallel measurements were conducted. User-friendly software was used to control the automated robot system, enabling a flexible movement of the FO-SPR probes and recording the obtained data.

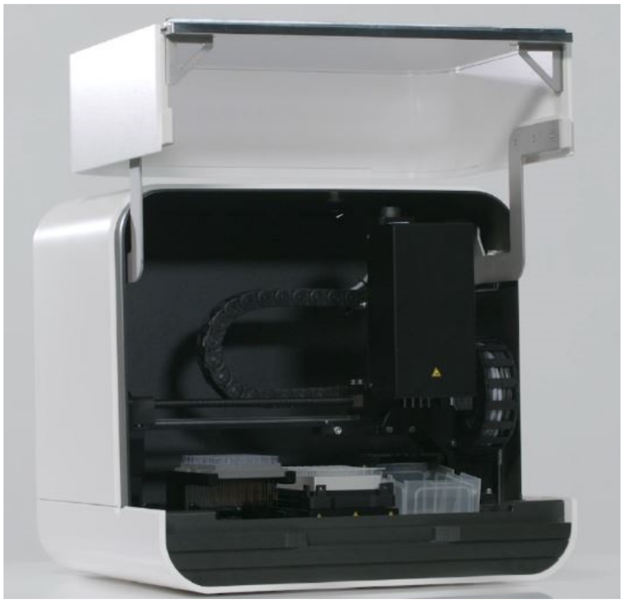

Figure S6. Picture of the FO-SPR platform (White FOx 1.0, commercialized by FOx Biosystems) used for EV analysis in this paper.

Reference

1. Geeurickx, E.; Tulkens, J.; Dhondt, B.; Van Deun, J.; Lippens, L.; Vergauwen, G.; Heyrman, E.; De Sutter, D.; Gevaert, K.; Impens, F.; et al. The Generation and Use of Recombinant Extracellular Vesicles as Biological Reference Material. *Nat. Commun.* **2019**, *10*, 1–12, doi:10.1038/s41467-019-11182-0.
